# *HER2*突变晚期非小细胞肺癌的靶向治疗进展

**DOI:** 10.3779/j.issn.1009-3419.2025.106.22

**Published:** 2025-08-20

**Authors:** Jinyu YU, Baoshan CAO

**Affiliations:** ^1^100191 北京，北京大学第三医院肿瘤化疗与放射病科（于金玉，曹宝山）; ^1^Department of Medical Oncology and Radiation Sickness; ^2^肿瘤中心（于金玉，曹宝山）; ^2^Cancer Center, Peking University Third Hospital, Beijing 100191, China

**Keywords:** 肺肿瘤, 人类表皮生长因子受体2, 突变, 靶向治疗, Lung neoplasms, Human epidermal growth factor receptor 2, Mutation, Targeted therapy

## Abstract

人类表皮生长因子受体2（human epidermal growth factor receptor 2, *HER2*）突变在非小细胞肺癌（non-small cell lung cancer, NSCLC）中发挥着驱动基因的作用。*HER2*突变的晚期NSCLC患者对传统化疗和免疫治疗的疗效不佳，因此靶向HER2的治疗正在进行广泛的研究。本综述分析了*HER2*突变在NSCLC中的生物学特性、靶向治疗药物-包括大分子单抗、酪氨酸激酶抑制剂（tyrosine kinase inhibitors, TKIs）和抗体偶联药物的临床研究概况以及耐药研究方向。吡咯替尼和德曲妥珠单抗目前获批用于治疗标准治疗失败后的*HER2*突变晚期NSCLC患者，但远不能满足临床需求，新型选择性HER2 TKIs逐渐崭露头角。未来的探索趋势正在从单一的药物逐渐转向联合策略，并且探索更为精准的选择策略以及耐药机制研究。这些研究将为*HER2*突变晚期NSCLC的临床治疗策略提供理论依据，推动个体化治疗的发展。

## Abstract (en)

Human epidermal growth factor receptor 2 (*HER2*) mutations play a role as a driver gene in non-small cell lung cancer (NSCLC). Patients with advanced NSCLC harboring *HER2* mutations exhibit poor responses to conventional chemotherapy and immunotherapy, hence targeted therapies against HER2 are under extensive investigation. This review analyzes the biological characteristics of *HER2*, an overview of clinical trials for targeted therapy drugs, including monoclonal antibodies, tyrosine kinase inhibitors (TKIs), and antibody-drug conjugate, and research directions for drug resistance in NSCLC. Currently, Pyrotinib and Trastuzumab deruxtecan have been approved for the treatment of advanced NSCLC with *HER2 *mutations, suitable for patients who have failed standard therapy, which is far from meeting the clinical demands. Novel selective HER2 TKIs are gradually emerging. Future exploration trends are gradually shifting from single drugs to combination strategies, and are exploring more precise selection strategies as well as research on resistance mechanisms. These studies will provide a theoretical basis for clinical treatment strategies for advanced NSCLC with *HER2 *mutations, promoting the development of personalized therapy.

肺癌是全球病死率最高的恶性肿瘤之一，其中非小细胞肺癌（non-small cell lung cancer, NSCLC）约占85% ^[1]^。近年来，随着对驱动基因的深入研究，靶向治疗在NSCLC中取得了显著进展，尤其是表皮生长因子受体（epidermal growth factor receptor, *EGFR*）突变的患者^[2]^。随着精准治疗理念的深入，少见靶点人类表皮生长因子受体2（human epidermal growth factor receptor 2, HER2）受到越来越多的关注，它的异常激活不仅是NSCLC的驱动基因，也是EGFR抑制剂耐药的主要原因之一^[3,4]^。HER2是受体酪氨酸激酶（receptor tyrosine kinase, RTK）家族的一员，与EGFR、HER3和HER4共同参与细胞生长、分化和转移的调控^[5]^。携带*HER2*突变的NSCLC患者，传统化疗中位无进展生存期（median progression-free survival, mPFS）仅5个月，无法满足临床治疗的需求^[6]^。尽管HER2靶向治疗领域取得了一定的进展，但仍面临诸多挑战。本文综述了*HER2*突变在NSCLC中的生物学特性和治疗进展，为未来精准治疗提供了重要参考。

## 1 HER2与NSCLC

HER2是HER家族的成员之一，其他成员包括HER1、HER3及HER4；与HER家族其他成员不同，HER2没有相应的配体，而是通过与自身或家族其他成员形成同源或异源二聚体，触发酪氨酸激酶残基磷酸化并激活下游信号通路，包括丝裂原活化蛋白激酶（mitogen-activated protein kinase, MAPK）及磷脂酰肌醇-3-激酶（phosphatidylinositol-3-kinase, PI3K）信号通路等，从而促进肿瘤的发生发展（图1）^[7-10]^。*HER2*的变异类型包括基因突变、扩增和过表达。NSCLC中1%-5%的患者携带*HER2*基因突变^[11,12]^。目前，小样本的研究^[13]^提示靶向HER2在HER2过表达的NSCLC中初见成效，但主要的研究进展仍集中在*HER2*突变的NSCLC中。

**图1:**
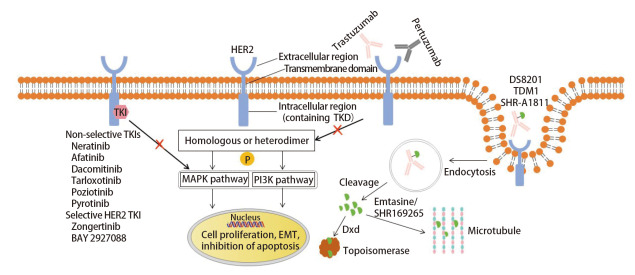
HER2下游信号通路及靶向HER2药物的作用机制

*HER2*突变患者通常发病年龄较轻，更常见于女性、非吸烟者以及肺腺癌患者，与较高的脑转移发生率有关，与*EGFR*、间变性淋巴瘤激酶（anaplastic lymphoma kinase, *ALK*）、Kirsten鼠类肉瘤病毒癌基因同源物（Kirsten rat sarcoma viral oncogene homolog, *KRAS*）、鼠类肉瘤滤过性毒菌致癌同源体B（v-Raf murine sarcoma viral oncogene homolog B1, *BRAF*）等驱动基因突变互斥^[14-17]^。大约90%的*HER2*突变是20外显子的非移码插入突变，主要的变异是重复出现的12个碱基对插入，最常见的是A775_G776insYVMA突变（YVMA），其次是G776delinsVC、P780_Y781insGSP和G776delinsLC^[16,18]^。*HER2*突变的NSCLC患者较其他驱动基因突变的患者对培美曲塞化疗的效果欠佳，其中YVMA插入突变患者化疗预后显著差于其他突变类型的患者；一线接受化疗联合免疫治疗患者的1年生存率也仅为52%^[6,19,20]^。因此，开发靶向HER2的治疗策略对*HER2*突变的晚期NSCLC患者至关重要。

## 2 NSCLC中靶向HER2突变的治疗

目前尚无专门针对*HER2*突变NSCLC的一线特异性靶向药物获批，靶向*HER2*突变NSCLC的药物正逐步开展广泛的研究，包括大分子抗HER2抗体、酪氨酸激酶抑制剂（tyrosine kinase inhibitors, TKIs）以及抗体偶联药物（antibody-drug conjugate, ADC）（图1，表1）。

**表1:** *HER2*突变NSCLC靶向HER2治疗的研究汇总

Drug	Study	Line of therapy	*HER2* mutation type	*n*	ORR (%)	mPFS (mon)	mOS (mon)	≥Grade 3 AEs and incidence	Reference
Trastuzumab+ Pertuzumab	Mypathway	≥2	Mutation	14	21	-	-	-	[21]
	DRUP	≥2	Ex20 mutation	24	8.3	4	10	-	[22]
Trastuzumab+ Pertuzumab+ Docetaxel	IFCT-1703 R2D2	≥2	Mutation	45	29	6.8	17.6	Gastrointestinal AEs 15.6%, fatigue 6.7%, skin AEs 11.1%, neurological AEs 6.7%, hematologic/lymphatic system 48.9%, increased transaminases 2.2%, respiratory system 2.2%	[23]
Pyrotinib	NCT02834936	≥2	Mutation	60	30	6.9	14.4	Diarrhea 20%, vomiting 1.7%, increased transaminases 1.7%	[24]
	NCT02535507	≥2	Ex20 insertion mutation	15	53.3	6.4	-	None	[25]
	NCT03574402	1 (CF)	Mutation	28	35.7	7.3	14.3	Increased aspartate aminotransferase 3.6%, increased alanine transaminase 3.6%, hyponatremia 3.6%, hypokalemia 3.6%	[26]
	NCT03574402	1 (CU)	Mutation	12	16.7	4.7	14.2	Diarrhea 16.7%, hyponatremia 8.3%, hypokalemia 8.3%, anemia 8%	[26]
	NCT03605602	1 (RWS)	Mutation	8	0	3	12.2	-	[26]
Pyrotinib+ Apatinib	PATHER2	≥2	Mutation (*n*=31), amplification (*n*=2)	33	51.5	6.9	14.8	Diarrhea 3%, hypertension 9.1%	[27]
Pyrotinib+ Inetetamab	NCT05016544	≥1	Mutation	41	26.8	5.5	-	Diarrhea 4.2%, increased serum creatinine 2.1%, paronychia 2.1%	[28]
Zongertinib	NCT04886804	≥1	TKD mutation	75	71	12.4	-	Diarrhea 1%, decreased neutrophil count 1%, increased transaminases 13%	[29]
		≥2	TKD mutation (previously treated with HER2 ADC)	31	48	-	-	Diarrhea 5%, increased transaminases 5%, drug-induced liver injury 10%	
		≥1	Non-TKD mutation	20	30	-	-	Nausea 3%	
BAY 2927088	SOHO-1	≥2	Mutation (previously treated with anti-HER2)	34	35.5	-	-	-	[30]
		≥2	Mutation	81	59.3	-	-	Diarrhea 23.5%, stomatitis 1.2%	[31]
		1	Mutation	39	59	-	-	Diarrhea 2.6%	[31]
Neratinib	SUMMIT	≥2	Mutation	26	3.8	5.5	-	Diarrhoea 22%, nausea 2.1%, vomiting 2.1%, constipation 1.4%, fatigue 3.5%, decreased appetite 0.7%, abdominal pain 5%, anaemia 7.1%, dyspnoea 3.5%, dehydration 5.7%, aspartate aminotransferase increased 3.5%, asthenia 0.7%	[32]
Afatinib	NICHE	≥2	Ex20 insertion mutation	13	7.7	4	14	Dyspnea 15.3%, 1 case of fatal acute renal failure, stomatitis, epistaxis, pleural effusion, increased γ-glutamyl transferase, electrolyte abnormalities, urinary tract obstruction, paraplegia, anemia, febrile neutropenia all <10%	[33]
	Fan Y et al. ^[34]^	≥2	Mutation	18	0	2.76	10.02	Diarrhea 16.7%, gastrointestinal discomfort 16.7%, infection 5.6%, increased transaminases 5.6%	[34]
Dacomitinib	NCT00818441	≥2	Mutation	26	12	3	9	Diarrhea 23%, dermatitis 3%, fatigue 3%	[35]
Tarloxotinib	RAIN-701	≥2	Mutation	11	22	-	-	QTc prolongation 34.8%, rash 4.3%, diarrhea 4.3%, increased transaminases 4.3%	[36]
Poziotinib	ZENITH20-2	≥2	Mutation	90	27.8	5.5	-	Rash 48.9%, diarrhea 25.6%, oral mucositis 24.4%, paronychia 1.1%, dry skin 5.6%, decreased appetite 2.2%, nausea 2.2%, pruritus 2.2%, fatigue 2.2%, anemia 3.3%, weight loss 1.1%, hypomagnesemia 2.2%, dyspnea 1.1%, asthenia 3.3%, hypokalemia 3.3%, hypocalcemia 2.2%, recurrent pancreatitis 1.1%, fatal pneumonia (*n*=1)	[37]
	ZENITH20-4	1	Mutation	80	39	5.6	-	Rash 42.5%, diarrhea 17.5%, oral mucositis 18.8%, paronychia 11.3%, dry skin 2.5%, decreased appetite 1.3%, altered taste 1.3%, pruritus 3.8%, fissured skin 2.5%, fatigue 3.8%, vomiting 2.5%, hypokalemia 6.3%, pneumonia 2.5% (including 1 case of fatal pneumonia)	[38]
	NCT03066206	≥2	Ex20 mutation	30	27	5.5	15	Rash 47%, diarrhea 20%, paronychia 20%, oral mucositis 10%, nausea 7%, weight loss 3%, anemia 7%, pruritus 7%, vomiting 3%, fatal pneumonia (*n*=1)	[39]
DS-8201	Destiny-Lung01	≥2	Mutation	91	55	8.2	17.8	Nausea 9%, fatigue 7%, vomiting 3%, neutropenia 15%, anemia 10%, diarrhea 2%, leukopenia 4%, fatal interstitial lung disease (*n*=1)	[15]
	Destiny-Lung02 (5.4 mg/kg)	≥2	Mutation	102	49	9.9	19.5	Nausea 4%, neutropenia 18.8%, fatigue 7.9%, decreased appetite 2%, anemia 10.9%, vomiting 3%, constipation 1%, leukopenia 5%, thrombocytopenia 5.9%, diarrhea 1%, increased transaminases 3%	[40]
DS-8201+ Pembrolizumab	DS8201-A-U106	≥2	Mutation	33	66.7	11.3	-	All ≥grade 3 AEs 48.5%, fatal interstitial lung disease (*n*=1)	[41]
TDM1	NCT02675829	≥1	Mutation	18	44	5	-	Anemia 6%	[42]
	JapicCTI-194620	≥2	Ex20 insertion	22	38.1	2.8	8.1	Thrombocytopenia 18.2%	[43]
SHR-A1811	HORIZON-Lung	≥2	Mutation	94	73	11.5	-	Neutropenia 41%, leukopenia 26%, anemia 23%, thrombocytopenia 11%, lymphopenia 7%, vomiting 2%, increased alanine aminotransferase 2%, increased GGT 2%, hyponatremia 2%, hypokalemia 3%, pneumonia 3%, fatigue, hypertriglyceridemia, gastritis, hypocalcemia, interstitial lung disease, diarrhea, decreased ejection fraction, small bowel obstruction, vulvitis all 1%	[44]

NSCLC: non-small cell lung cancer; ORR: objective response rate; mPFS: median progression-free survival; mOS: median overall survival; ADC: antibody-drug conjugate; Ex20: exon 20; CF: criteria-fulfilled; CU: compassionate use; RWS: real-world study; AEs: adverse events; GGT: glutamyl transpeptidase.

### 2.1 单克隆抗体

曲妥珠单抗和帕妥珠单抗是两种靶向HER2的大分子单克隆抗体药物，在*HER2*突变的NSCLC中疗效有限。Mypathway篮子研究^[21]^中，曲妥珠单抗联合帕妥珠单抗治疗的14例*HER2*突变NSCLC的客观缓解率仅为21%，DRUP研究^[22]^中双靶方案治疗*HER2 *20外显子突变的晚期NSCLC患者客观缓解率（objective response rate, ORR）仅为8.3%，mPFS和中位总生存期（median overall survival, mOS）分别为4和10个月，提示双靶方案治疗*HER2*突变的NSCLC疗效不尽人意。IFCT-1703 R2D2研究^[23]^在双靶的基础上联合了多西他赛治疗*HER2*突变的晚期NSCLC患者，结果显示ORR为29%，mPFS为6.8个月，mOS为17.6个月，该方案虽然在ORR的提高上并不明显，但在延长PFS和OS上看到了初步的效果。有待更大样本量研究验证双靶联合化疗作为HER2突变晚期NSCLC的潜能。

### 2.2 TKIs

靶向HER2的TKIs临床研究包括泛HER抑制剂及选择性HER2抑制剂，目前较为有前景的药物有吡咯替尼、Zongertinib以及BAY 2927088；而来那替尼、阿法替尼、达克替尼、溴拉替尼以及波齐替尼等药物因前期疗效欠佳或毒性过大已终止于早期研究。

#### 2.2.1 吡咯替尼

吡咯替尼在*HER2*突变的NSCLC中开展了单药及联合用药研究。在一项II期临床试验^[24]^中，吡咯替尼在经治的*HER2*突变NSCLC患者中显示出30%的ORR，其中某些特定突变亚型（如A775_G776insYVMA和G776_V777.VCV）表现出更高的ORR，mPFS为6.9个月，mOS为14.4个月；常见的3级及以上治疗相关不良事件（treatment-related adverse events, TRAEs）包括腹泻、皮疹和口腔炎，但多数情况下患者能够良好耐受。另一项只纳入了*HER2* 20外显子插入突变的NSCLC的II期研究^[25]^显示，15例经治患者接受吡咯替尼靶向治疗，ORR达53.3%，mPFS为6.4个月。基于上述研究结果，自2023年开始，吡咯替尼成为《中国临床肿瘤学会非小细胞肺癌诊疗指南》对晚期*HER2*突变的NSCLC二线治疗的推荐用药。在一线用药方面，一项II期研究^[26]^显示，在严格入组队列、同情给药队列和真实世界队列中，ORR分别为35.7%、16.7%和0%，mPFS分别为7.3、4.7和3.0个月，mOS分别为14.3、14.2和12.2个月。严格入组队列数据表明吡咯替尼的一线治疗*HER2*突变NSCLC患者的疗效确切；同情给药队列数据表明临床试验常规排除的NSCLC患者也可从吡咯替尼治疗中获益；由于真实世界给药组仅有8例患者，真实世界的疗效可能还需更大规模样本量观察。目前正在进行的III期临床试验（Pyramid-1, NCT04447118）将进一步评估吡咯替尼在*HER2*突变NSCLC患者中与多西他赛相比的疗效和安全性。

联合用药方面，PATHER2研究^[27]^采用吡咯替尼与阿帕替尼联合治疗*HER2*突变的NSCLC，33例可评估的患者ORR达到了51.5 %，mPFS为6.9个月，mOS为14.8个月，该研究的亚组分析提示，不论是否合并脑转移以及治疗的线数如何，患者均能从该方案中获益。吡咯替尼联合人源化抗HER2单抗伊尼妥珠单抗的Ib期研究^[28]^结果显示ORR为26.8%，mPFS为5.5个月，似乎并没有带来更有优势的结果。吡咯替尼联合培美曲塞加卡铂（NCT04706949）、程序性死亡受体1（programmed cell death protein 1, PD-1）抑制剂（NCT04144569）或抗血管生成药物沙利度胺治疗*HER2*突变的晚期NSCLC的研究也正在进行中，旨在寻找协同增效的更优方案^[45]^。

#### 2.2.2 Zongertinib

Zongertinib（BI 1810631）是一种不可逆的选择性HER2 TKI，它能够高度特异性地抑制HER2信号通路，避免对野生型EGFR的干扰，从而降低了EGFR相关毒性反应^[46]^。Beamion LUNG-1研究^[29]^显示，携带*HER2*酪氨酸激酶结构域（tyrosine kinase domain, TKD）突变的晚期NSCLC患者接受Zongertinib的ORR达71%，mPFS达12.4个月，常见突变亚型YVMA的ORR高达81%，并且在抗HER2 ADC经治的队列中，ORR仍能达到48%；而非TKD突变的患者ORR仅为30%，提示Zongertinib在*HER2* TKD突变的患者中有更为突出的疗效，并且可能成为HER2 ADC耐药后的一项重要治疗选择。AEs方面，腹泻和肝损伤是最常见的，无治疗相关性间质性肺疾病报道^[29]^。Zongertinib正在进行另一项III期BEAMION LUNG-2临床研究（NCT06151574），旨在将Zongertinib向一线治疗推进。

#### 2.2.3 BAY 2927088

BAY 2927088是一种口服可逆性HER2 TKI。SOHO-1研究^[30,31,47]^显示在晚期*HER2*突变NSCLC患者中，无论是初治还是经治的患者，BAY 2927088的ORR均为59%，值得注意的是携带YVMA突变的患者ORR高达90.0%，但是既往接受过抗HER2治疗的患者ORR降至35.5%。腹泻是最常见的AE，但是可控且未导致治疗中断^[30,31]^。提示BAY 2927088在未经抗HER2治疗的*HER2*突变NSCLC中，尤其是TKD突变患者中有着良好的治疗前景。随后正在进行的III随机对照研究SOHO-02研究^[48]^旨在评估BAY 2927088作为一线治疗在*HER2* TKD突变的局部晚期或转移性NSCLC患者中的疗效和安全性。

#### 2.2.4 其他TKIs

在*HER2*突变的NSCLC治疗中来那替尼单药效果不佳，但与恩美曲妥珠单抗（Trastuzumab emtansine, TDM1）联用有提高疗效的潜力^[32,49]^。阿法替尼在*HER2* 20外显子突变患者中mPFS约4个月，mOS约14个月，需进一步筛选靶点^[33,34]^。达克替尼ORR为12%，mPFS和mOS分别为3和9个月，整体疗效有限，且AEs较为常见，限制了其在*HER2*突变的晚期NSCLC的进一步应用^[35]^。溴拉替尼ORR仅为22%，研究已中止^[36]^。波齐替尼的ORR为27%-39%，但3/4级TRAEs发生率高，导致减量治疗，且因毒性问题未获美国食品药品监督管理局（Food and Drug Administration, FDA）批准^[37-39]^。

### 2.3 ADC

ADC是一种新型的靶向治疗药物，由单克隆抗体、药物分子以及连接这两个部分的化学连接子组成。这种药物的设计旨在将具有细胞毒性的药物分子直接递送到肿瘤细胞，从而提高治疗效果并减少对正常细胞的损害。ADC也在*HER2*突变的NSCLC治疗中显示出潜力。

#### 2.3.1 德曲妥珠单抗（Trastuzumab deruxtecan, DS-8201）

DS-8201由人源化抗HER2抗体、可裂解连接子和拓扑异构酶I抑制剂Dxd组成^[50]^。在DESTINY-Lung01^[15]^和DESTINY-Lung02^[40]^研究中，德曲妥珠单抗表现出对*HER2*突变NSCLC患者的高缓解率和持久的疗效。在II期临床研究DESTINY-Lung01^[15]^中，91例经治*HER2*突变NSCLC患者接受DS-8201治疗，ORR为55%，mPFS为8.2个月，mOS为17.8个月。然而，该项研究中3级及以上TRAEs的发生率为46%，包括1例致死性间质性肺炎，药物安全性引起广泛重视。因此，在随后开展的DESTINY-Lung02研究^[40]^中，对DS-8201的安全剂量进一步探索，结果发现5.4 mg/kg剂量组的安全性比6.4 mg/kg剂量组更好，尤其是间质性肺炎的发生率更低，而两个剂量组的ORR无明显差异。基于DESTINY-Lung02研究的结果，FDA于2022年8月加速批准了DS-8201用于治疗*HER2*突变的不可切除或转移性NSCLC患者，并且也被美国国立综合癌症网络（National Comprehensive Cancer Network, NCCN）指南及《中国临床肿瘤学会非小细胞肺癌诊疗指南》推荐用于治疗*HER2*突变晚期NSCLC的二线及以上治疗。目前，DESTINY-Lung04研究（旨在评估DS-8201在携带*HER2* 19或20外显子突变的局晚或晚期NSCLC患者中一线治疗的疗效和安全性）和DESTINY-Lung05研究（旨在中国评估DS-8201在携带*HER2* 19或20外显子突变二线及以上的转移性非鳞NSCLC患者中的疗效和安全性）正在进行中，为治疗前移及在中国取得适应证提供依据。

#### 2.3.2 恩美曲妥珠单抗

TDM1由曲妥珠单抗与抗微管药物emtasine偶联而成^[51]^。一项II期篮子试验^[42]^显示，TDM1用于接受过多线治疗的*HER2*突变的NSCLC患者，ORR为44%，mPFS为5个月，AEs主要是1-2级；不同突变亚型的ORR存在差异：*HER2* 20外显子插入突变为55%，其他亚型为29%，19外显子突变无反应。随后日本的一项II期研究^[43]^纳入了*HER2* 20外显子插入突变的NSCLC患者，其中YVMA最常见（86.4%），ORR、mPFS和mOS分别为38.1%、2.8和8.1个月。这提示*HER2*突变NSCLC人群中可能存在异质性，患者的选择需要更为精准的标志物。目前TDM1被NCCN指南推荐用于*HER2*突变的NSCLC的后线治疗，推荐级别低于DS-8201。

#### 2.3.3 SHR-A1811

SHR-A1811由曲妥珠单抗与拓扑异构酶I抑制剂（SHR169265）偶联。II期研究HORIZON-Lung^[44]^中，纳入的94例HER2突变的NSCLC患者，ORR为73%，PFS为11.5个月，46.0%的患者出现了3级及以上的TRAEs，最常见的3级及以上TRAEs为血液学毒性，未出现致死性治疗相关的间质性肺疾病。基于*HER2*突变蛋白功能变化以及*HER2*突变NSCLC免疫抑制的微环境特点，SHR-A1811联合吡咯替尼/PD-1单抗（SHR-1316）（NCT05482568）也在开展中。

### 2.4 抗HER2联合免疫治疗

HER2突变的NSCLC具有一些独特的肿瘤微环境（tumor microenvironment, TME）特征，主要包括：细胞程序性死亡-配体1（programmed cell death ligand 1, PD-L1）表达水平较低、肿瘤突变负荷低、干扰素基因刺激蛋白通路的抑制以及颗粒酶和穿孔素表达水平低^[52-55]^。以上造就了*HER2*突变NSCLC免疫抑制的微环境，可能是其对免疫治疗效果欠佳的原因，但同时也为抗HER2治疗联合免疫治疗提供了依据。吡咯替尼、SHR-A1811分别联合PD-1抑制剂（NCT04144569）（NCT05482568）的研究正在进行中。临床前研究^[56]^提示DS-8201可增强T细胞的活性并上调PD-L1的表达，与PD-1治疗存在协同作用。DS8201-A-U106研究^[41]^中采用5.4 mg/kg剂量的DS-8201联合帕博利珠单抗治疗*HER2*突变的NSCLC，在ORR和PFS上较DS-8201单药似乎更优，但是48.5%的患者出现了3级及以上的TRAEs，其中包括1例致死性间质性肺炎，提示ADC药物联合免疫治疗的安全性需更多地关注。在NSCLC中PD-L1单抗的AEs发生率显著低于PD-1单抗^[57]^。HUDSON研究旨在评估DS8201联合度伐利尤单抗的疗效与安全性尚未有结果报道，值得继续关注。

### 2.5 耐药研究进展

与其他靶向治疗一样，抗HER2靶向治疗的耐药也是不可避免的，但是目前尚缺乏大样本的数据来明确肺癌中的耐药机制。NSCLC与其他癌种的*HER2*变异谱及药物敏感性不同，不能照搬其他肿瘤的耐药机制。既往在乳腺癌的研究^[58-60]^中发现，HER2水平的下降或该受体的结构改变可能是抗HER2 ADC耐药的原因。但在*HER2*突变的NSCLC中尚未发现HER2的表达与抗HER2 ADC药物之间有相关性。理论上，参考同家族NSCLC中常见的驱动基因*EGFR*的TKIs耐药机制，抗HER2耐药可能通过HER2依赖性和非依赖性途径发生，包括*HER2*的二次突变和旁路激活，如RAS/MAPK信号通路或PI3K/蛋白激酶B（protein kinase B, AKT）通路等^[7-9]^。而现有研究^[61]^发现吡咯替尼的耐药主要是上调的上皮间质化和血管生成通路，这与参考EGFR的理论推测并不一致。因此，NSCLC的抗HER2治疗耐药机制需要更多的源头研究。小分子TKIs方面，现有的研究^[62,63]^结果提示，不同*HER2*突变亚型对小分子TKIs的敏感性不同，通过计算模拟和分子动力学模拟，研究者们分析了*HER2*突变对药物结合位点的影响，发现YVMA插入突变由于其空间位阻，对某些HER2靶向药物（如阿法替尼）表现出较低的敏感性，而G776delinsVC和G778_P780dup对阿法替尼及吡咯替尼表现出较高的敏感性。此外，药物结合模式、突变位置都可能会对TKIs药物敏感性产生不同的影响^[62-64]^。在抗HER2 ADC方面，研究也发现不同突变亚型对药物的敏感性似乎不同，基础研究^[49]^提示，这可能与不同突变亚型介导受体内化相关。此外，大分子单抗药物部分通过抗体依赖的细胞介导的细胞毒作用效应发挥作用，前文中提到的*HER2*突变NSCLC的免疫抑制的微环境也可能影响靶向治疗的效果^[65]^。上述内容为NSCLC抗HER2治疗耐药研究提供了方向，但是远远不能展示耐药机制全貌，作为NSCLC精准治疗的重要手段，阐明耐药机制为后续探索精准选择和联合用药具有重要意义，针对NSCLC的抗HER2治疗耐药的原创性研究尤为重要。

## 3 总结与展望

本文综述了*HER2*突变在晚期NSCLC的生物学特性及靶向治疗的最新进展。*HER2*突变的NSCLC患者通常具有独特的临床和分子特征，且对传统化疗及免疫治疗的反应较差，精准的靶向治疗策略的发展为*HER2*突变NSCLC提供了新的治疗选择，特别是TKIs吡咯替尼、Zongertinib、BAY 2927088和ADCs DS-8201、SHR-A1811，在临床试验中显示出了一定的疗效，曲妥珠单抗联合帕妥珠单抗和多西他赛也是潜在的治疗方案。将后线疗效确切的药物及时推向一线治疗有望为患者提供更好的临床获益，关键III期一线研究是推动治疗格局变革的核心动力，更大的样本量可以推动药物精准选择，例如细分脑转移亚组、不同突变类型群体的药物选择；以及为毒性管理提供更多经验。面对众多新型药物，精准的个体化治疗成为提高临床疗效的重要手段。结合现有数据，个体化治疗的选择主要依赖于肿瘤的分子特征以及患者的合并症情况。选择性HER2 TKIs对TKD突变似乎有着更好的疗效；而不同类型的抗HER2药物存在着不同的AEs谱（图2），ADC药物的AEs主要集中在血液学毒性，TKIs类药物则主要集中在消化道反应和皮肤毒性。联合用药是未来重要的探索方向，针对同一靶点的不同作用原理的药物联合具有创新性与引领性，正在开展的研究包括TKIs联合化疗、ADC联合免疫、ADC联合TKIs、TKIs联合免疫，联合用药是否优于单药的效果需后续的随机对照试验进行验证；毒性方面ADC联合免疫可能会导致间质性肺炎的毒性叠加，ADC联合TKIs或TKIs联合免疫可能导致腹泻和间质性肺疾病的毒性叠加，毒性管理或将是联合用药研究的一项重要任务。

**图2:**
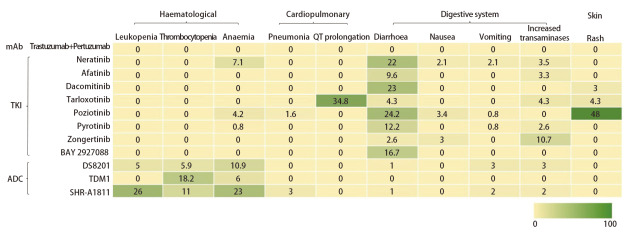
药物常见的≥3级以上不良反应分布。 DS-8201采用了Destiny Lung02研究5.4 mg/kg组的数据，TKIs和ADC药物均采用单药治疗数据，如有多个研究同时报道同予合并计算；表格中的数字代表不良反应发生率（%）。

未来的研究需要集中在以下几个关键领域：（1）精准筛选，发掘更为精确和高效的标志物，以便更好地筛选出适合靶向治疗的患者；（2）个体化治疗策略，基于患者的具体突变类型和分子特征，结合患者合并的基础病，制定个性化的治疗方案，以提高治疗效果并减少AEs；（3）联合治疗探索，研究不同药物组合的协同效应，采用联合用药的方法提高疗效并推迟耐药的发生，合理的序贯治疗可能降低毒性；（4）耐药机制研究，深入研究*HER2*突变NSCLC的耐药机制，识别新的生物标志物，并开发针对性的克服耐药的治疗策略。随着对*HER2*突变NSCLC分子机制理解的不断深入，结合新型药物的开发和现有治疗方法的优化，我们有理由相信，未来将能够为*HER2*突变的NSCLC患者提供更为有效和个性化的治疗方案。
